# Viral Circular RNAs and Their Possible Roles in Virus-Host Interaction

**DOI:** 10.3389/fimmu.2022.939768

**Published:** 2022-06-17

**Authors:** Xing Zhang, Zi Liang, Chonglong Wang, Zeen Shen, Sufei Sun, Chengliang Gong, Xiaolong Hu

**Affiliations:** ^1^ School of Biology and Basic Medical Science, Soochow University, Suzhou, China; ^2^ Institute of Agricultural Biotechnology and Ecological Research, Soochow University, Suzhou, China

**Keywords:** circRNA, host-virus interactions, viral infection, DNA viruses, RNA viruses

## Abstract

Circular RNAs (circRNAs) as novel regulatory molecules have been recognized in diverse species, including viruses. The virus-derived circRNAs play various roles in the host biological process and the life cycle of the viruses. This review summarized the circRNAs from the DNA and RNA viruses and discussed the biogenesis of viral and host circRNAs, the potential roles of viral circRNAs, and their future perspective. This review will elaborate on new insights gained on viruses encoded circRNAs during virus infection.

## Introduction

Viruses are a major class of intracellular parasites that use the transcription and translation machinery of the host to produce nucleic acids and proteins for their life cycle and reproduction. Virally infected hosts suffer immunosuppression, development disorder, and loss of energy and nutrients, causing diseases. Another subset of viruses can mutually benefit the hosts that will not cause disease (1). These virus-host interactions may appear by coevolution for more than thousands of years. Hosts have evolved various antiviral immune protection mechanisms due to the selection pressure caused by viral infection. At the same time, viruses have developed a variety of evasion defenses, including innate and adaptive immunity for their reproduction (2, 3). In the interaction between viruses and hosts, a variety of regulatory molecules have evolved from multiple mechanisms, which include interferon regulatory factors (4), antisense RNAs (5), microRNAs (6), and antiviral peptides (7).

**Figure 1:**
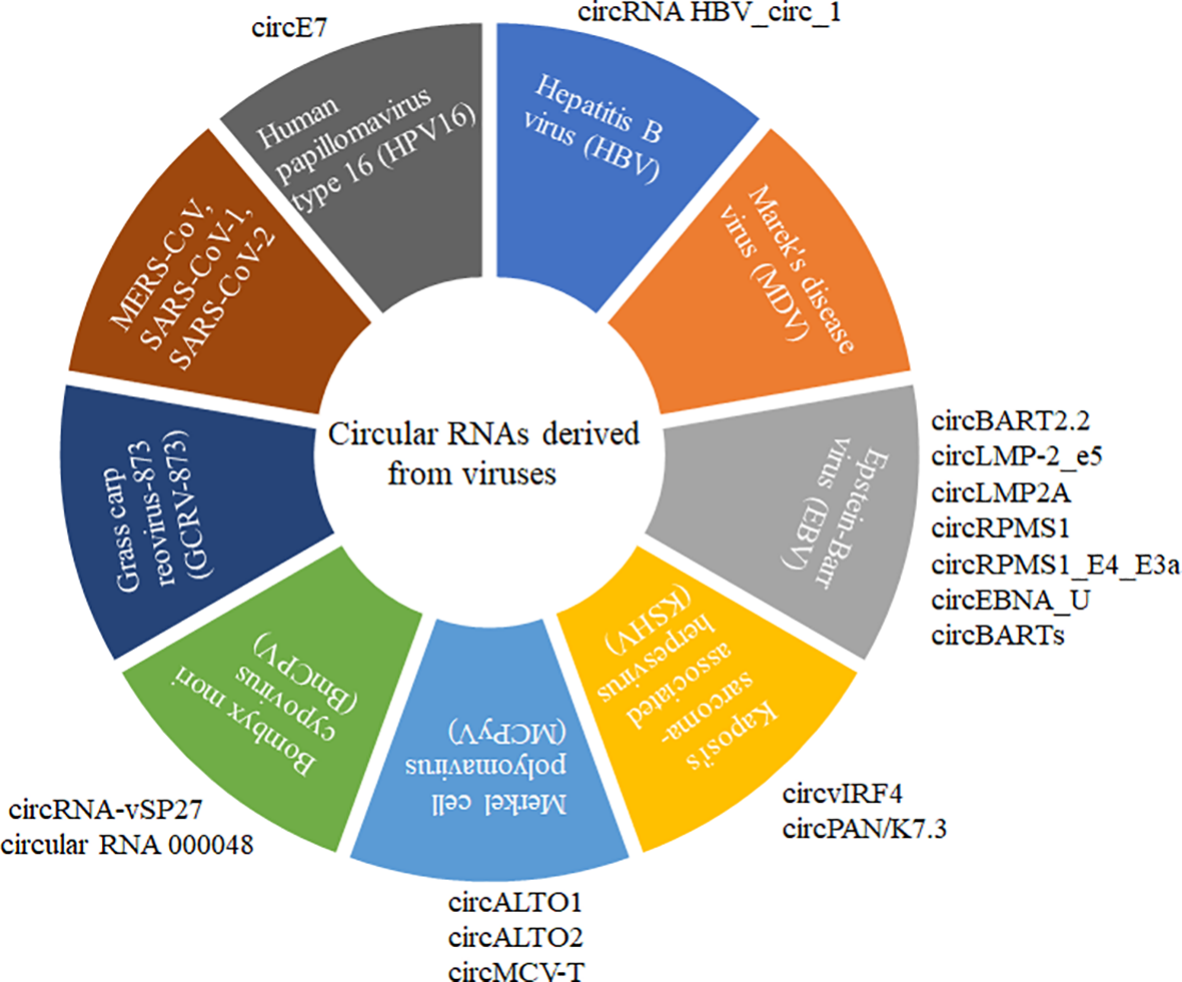
CircRNAs derived from different DNA and RNA viruses with various genome types. MERS-CoV, SARS-CoV-1, SARS-CoV-2, GCRV-873, and BmCPV are RNA viruses. HPV16, HBV, MDV, EBV, KSHV, and MCPyV are DNA viruses. MERS-CoV, SARS-CoV-1, and SARS-CoV-2 belong to Coronaviridae with positive sense and single-strand RNA genome. GCRV-873 and BmCPV belong to Reoarviridae with a double-strand RNA genome. HPV16, HBV, MDV, EBV, KSHV, and MCPyV with double-strand DNA genomes are closely related to the occurrence of tumors or cancers.

Circular RNA (circRNA) is a recently discovered functional non-coding RNA molecule, covalently closed circular single-stranded RNA molecules without a 5’-terminal cap structure and a 3’-terminal polyadenylate (8, 9). circRNA with a stable structure is resistant to degradation by exonuclease R and has Spatio-temporal and tissue expression specificity (10–12). Before 2013, circRNA was always considered the byproduct of abnormal RNA splicing without regulatory function. However, increasing evidence showed that various circRNAs commonly existed in natural organisms with conservative sequences among different species. Until now, the identification of circRNAs and their possible roles have been explored in more than 20 species, including Archaea (8), plants (11, 13, 14), viruses (15–19), insects (20, 21), and mammals (12, 22, 23). A small subset of circRNAs with different activities was confirmed to play vital roles in regulating individual growth and development, environmental stress, innate immunity, and disease occurrence (13, 24–28). Normally, circRNAs are often ignored by their low expression pattern and non-ploy (A) tail in the experiment. Recently, virally encoded circRNAs were reported from more than 10 different viruses with various genome types (**Figure 1**).

This review will concentrate on circRNAs in viruses, focusing on classes and features of virally circRNAs, the potential functions of virally circRNAs, and possible biogenesis mechanisms, providing clues to explore better the roles in the interplay between viruses and hosts.

## Virally circRNAs Encoded by DNA Viruses

DNA viruses with a relatively larger and more stable DNA genome mainly include three categories according to the types of their genome: double-stranded DNA viruses, single-stranded DNA viruses, and pararetroviruses (29). Genomes of DNA viruses have a wide size range from less than 2 kb to over 375 kb. It is noted that DNA viruses can be separated into early and late phases based on the transcription of viral genes before DNA synthesis (30). Host transcription and translation systems were involved in the viral mRNAs transcription and viral proteins production. Most DNA viruses are intranuclear replicating viruses, but only a small fraction of the virus (poxviruses) is entirely replicated in the cytoplasm (31). The most widely studied DNA viruses encoded circRNAs were related to the disease pathogenesis by acting as miRNA or RNA-binding protein sponge, oncoprotein translation, transcriptional regulators, and mRNA trap (19, 32–37).

### Human Papillomaviruses (HPVs) Encoded circRNAs

HPVs with small double-stranded DNA genomes can infect stratified epithelia. It is noted that most the HPV infections were not brought risks to the infected individuals with asymptomatic or caused benign warty, and only a small subset of ‘high-risk’ HPV infected individuals developed into cancers based on the infection sites (19). circRNA (circE7) encoded by Human papillomavirus type 16 (HPV16) could be identified in two HPV16-positive cervical cancer cell lines (CaSki and SiHa), and circE7 was formed *via* the pre-mRNA back-splicing of E6E7 (35). HPV16 circE7 with N6-methyladenosine (m6A) modification is predominantly distributed in the cytoplasm, translating E7 oncoprotein (19). These circRNAs derived from HPV were demonstrated to have translation activity and could be modified by the host cellular RNA methyltransferase. Viral circRNAs, viral circRNAs translated proteins, and m6A modified circRNAs play vital roles in mediating the infection, latency, and tumorigenesis.

### Hepatitis B Virus (HBV) Encoded circRNAs

HBV with partially double-stranded relaxed circular DNA genome converted into the covalently closed circular DNA following HBV infection. circRNA HBV_circ_1 was confirmed and generated by HBV and could be detected in HBV-positive HepG2.2.15 cells and HBV-related hepatocellular carcinoma tissue. The interaction between HBV_circ_1 and cyclin-dependent kinase 1 played pivotal roles in the cell proliferation, and the ectopic HBV_circ_1 expression in node mice could stimulate the tumor growth (37). RNA binding factor DExH‐Box helicase 9 (DHX9) bound to the inverted repeat sequences flanking the HBV pgRNA that inhibited the viral circRNA biogenesis, and this host protein DHX9 may be a novel regulator of the expression levels of viral circRNA and viral protein (38).

### Marek’s Disease Virus (MDV) Encoded circRNAs

MDV infection cause Marek’s disease (MD), which widespread induces T-cell lymphomagenesis in the domestic chicken. A large variety of MDV circRNAs was identified through RNA-sequencing, and the hot spots of circRNAs expression occurred on the major viral oncogenes in herpesviruses. Moreover, many noncanonical junction sites were observed in viral circRNAs compatible with the U2-dependent splicing machinery (39).

### Epstein-Barr Virus (EBV) Related circRNAs

EBV was identified as the first human tumor virus, closely related to the occurrence and development of gastric carcinoma (GC), nasopharyngeal carcinoma (NPC), and several lymphomas. CircBART2.2 derived from EBV could increase the expression level of PDL-1 in NPC and dysregulate the function of T-cells, leading to the tumor immune escape by multiple biological processes (33). EBV circLMP-2_e5 formed from the fifth exon of the *LMP-2* gene could be universally detected in EBV-positive cell lines. The expression pattern of circLMP-2_e5 was consistent with its linear parental transcript following EBV lytic reactivation. circLMP-2_e5 may be generated by exon skipping because none of the cis-elements were found in the short flanking introns (40). EBV circLMP2A induced and maintained the stemness phenotypes by the competing endogenous RNA (ceRNA) mechanism of the miR-3908/TRIM59/p53 signaling pathway, and its high expression level was associated with EBV-associated GC (34). EBV-derived circRPMS1 was associated with a short survival time, and the reduced expression level suppressed NPC proliferation and metastasis (41). EBV circ_RPMS1 localized in cytoplasm and nuclei is derived from the exons 2-4 of its *RPMS*1 gene (42). CircRPMS1_E4_E3a and circEBNA_U were revealed in rhesus macaque lymphocryptovirus orthologues of the latency-associated EBV. CircBARTs were expressed in EBV-positive patients’ tissues and cells. Two EBV circRNAs derived from the RPMS1 locus were detected in EBV-positive clinical stomach cancer specimens (17).

### Kaposi’s Sarcoma-Associated Herpesvirus (KSHV) Related circRNAs

Kaposi’s sarcoma-associated herpesvirus (KSHV) is an oncogenic γ2 herpesvirus, also called human herpesvirus-8 (HHV-8) (43). It is closely linked to primary effusion lymphoma, multicentric Castleman’s disease, and Kaposi’s sarcoma (44). CircvIRF4 was constitutively expressed in virally infected patients (16). CircvIRF4 generated by KSHV viral interferon regulatory factor 4 (vIRF4) was highly expressed in the KSHV-positive patient and cell lines (45). KSHV-derived circRNA could be incorporated into virions, while the viral circRNA produced on polyadenylated nuclear (PAN) RNA/K7.3 locus was found with its expression level paralleled with the linear transcript (46). Multiple viral circRNAs derived from the KSHV genome were revealed in KSHV-positive cells and patients (15). KSHV circvIRF4 was identified as the predominant viral circRNA (47). These viral circRNAs derived from the HSKV genome may be closely linked to the occurrence and development of related cancers. MDV, EBV, and KSHV-associated herpesvirus were related to the development of lymphoma, and the viral circRNAs encoded by these viruses were comprehensively reported by different groups.

### Merkel Cell Polyomavirus (MCPyV) Encoded circRNAs

Two viral circALTO1 and circALTO2 encompassing the complete gene of alternative large T antigen open reading frame (ALTO) were encoded by Merkel Cell Polyomavirus (MCPyV), and they could also be detected in the virus-positive cells and patients’ tissues. In addition, the related *Trichodysplasia spinulosa* polyomavirus (TSPyV) also encoded a circALTO, which existed in the virally infected tissues and cell lines (48). MCPyV encoded four circRNAs in its early region by detecting RNase R-resistant RNA sequencing. circMCV-T, with the most abundant expression level, was formed by back-splicing all early region exon II (49). Viral circRNAs are widespread in various DNA virus-infected individuals or cell lines, and their generation has been confirmed as key regulators in virus-induced cancers and tumorigenesis.

## Virally circRNAs Encoded by RNA Viruses

RNA viruses are normally recognized as very simple entities with small genomes, and the size of the length is from less than 2 to 32 kb. A relatively small genome of these RNA viruses has been confirmed with limited coding capacity. They are obligate intracellular parasites like DNA viruses. Host cells provide the energy, ribosome, deoxyribonucleotides, transcription and translation machinery, and other components that play important roles in assembling and releasing progeny virus (50). The genome types of RNA viruses have single-stranded (ssRNA) and double-stranded (dsRNA) nucleic acid. RNA viruses with a high mutation rate for the variation of the RNA genome that enable them to survive the varied environments during their life cycles (50, 51). Only several RNA viruses have been reported to encode viral circRNAs, including members from Reoarviridae (36, 52, 53) and Coronaviridae (54, 55).

### Reoarviridae Related circRNAs

*Bombyx mori* cytoplasmic polyhedrosis virus (BmCPV) is one of the most serious pathogens in the sericulture and bright huge economic loss to the farmers. This virus with 10 segmented dsRNA genome can specifically infect the midgut of the silkworm. Until now, the interaction between BmCPV and silkworm are poorly understood. circRNAs was considered a novel type of regulators that can regulate multiple biological processes of viruses or host. BmCPV derived circRNA-vSP27 have translation activity, and its translation production vSP27 small peptide suppressed viral replication *via* ROS-NF-κB signaling pathway, and was recruited with nuclear factor Akirin for activating NF-κB signaling pathway (53) as well as vsp21 was translated from the BmCPV circular RNA 000048 and attenuated viral replication (36). In addition, Grass carp reovirus (GCRV) is also a member of Reoarviridae with a segmented dsRNA genome containing 11 segmented dsRNAs. Infection of GCRV leads to grass carp hemorrhagic disease, one of the most serious diseases in the grass carp aquaculture industry (52, 56). In the GCRV-873 strain infected kidney cell line CIK, 32 circRNAs were identified, and some were confirmed to mediate the viral proliferation (52).

### Coronaviridae Related circRNAs

Coronaviruses MERS-CoV, SARS-CoV-1, and SARS-CoV-2 (SARS-CoV-1/2) have brought huge threating to our life and cause enormous morbidity and mortality to humans (54). MERS-CoV, SARS-CoV-1, and SARS-CoV-2 were identified to produce multiple viral circRNAs with low expression levels, but these viral circRNAs played important roles in several biological processes (54). Two major back-splice events were found among these viruses, and coronavirus produced more abundant and longer than their host circRNAs (55). It is noted that major dsRNA viruses have only exons and the viral circRNAs derived from these viruses have noncanonical splicing mechanisms. The viral transcripts circularization still needs more exploration by increasing groups.

## The Features of Virally circRNAs

**Figure 2:**
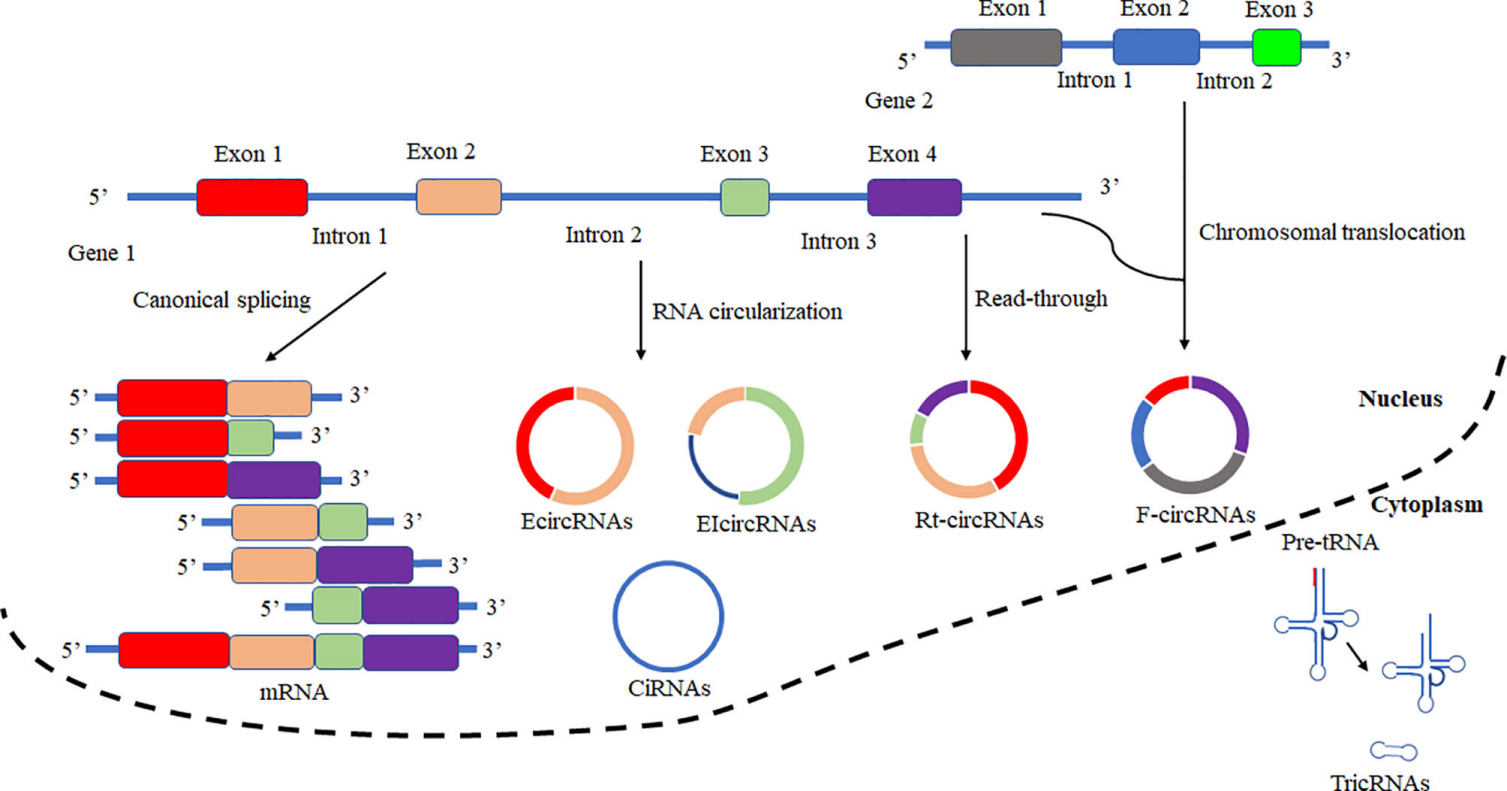
Schematic presentation of circRNAs biogenesis and canonical alternative splicing. CircRNAs are classified into six categories according to the formation types. Exonic circRNAs (EcircRNAs), Circular intronic circRNAs (CiRNAs), Exon-intron circRNAs (EIcircRNAs), Fusion circRNAs (F-circRNAs), Read-through circRNAs (Rt-circRNAs), and noncanonical circRNAs like tricRNAs. In canonical alternative splicing, consecutive or selected exons are joined together to generate a series of linear mRNAs to be subsequently translated.

**Figure 3:**
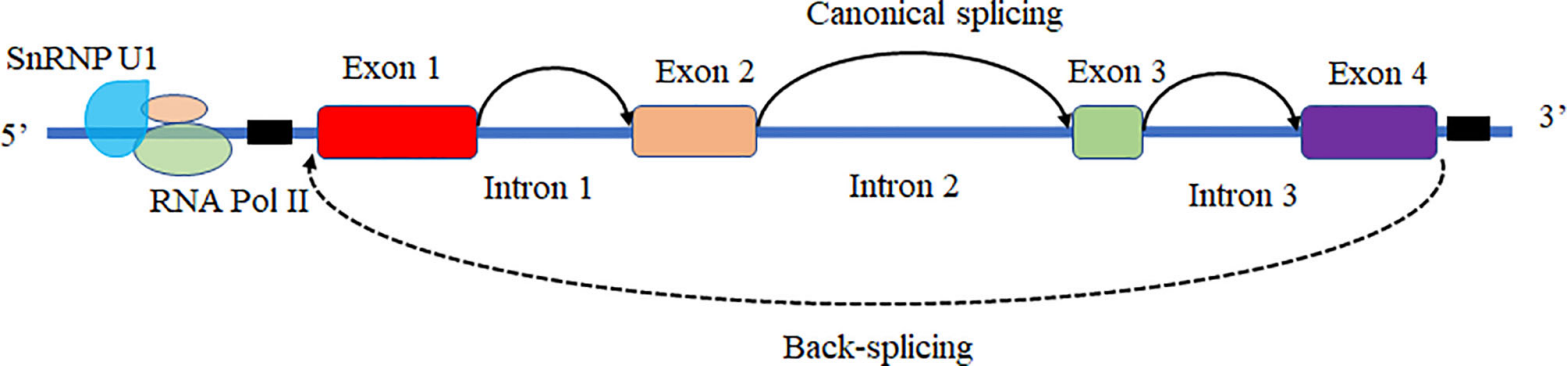
The mechanism of back-splicing and alternative splicing of the pre-mRNA. CircRNA is formed by a 3′,5′ phosphodiester bond using the downstream 5′ splice site (A5SS) and an upstream 3′ splice site (A3SS). Alternative splicing linear RNAs are formed by a 3′,5′ phosphodiester bond using an upstream 5′ splice site (A5SS) and a downstream 3′ splice site (A3SS).

Based on the biogenesis of circRNAs in a eukaryote, the formation of circRNAs is classified into six categories: exonic circRNAs (EcircRNAs), circular intronic cirRNAs (CiRNAs), exon-intron circRNAs (EIcircRNAs), fusion circRNAs (f-circRNAs), read-through circRNAs (rt-circRNAs) and noncanonical circRNAs like tricRNAs (**Figure 2**) (57). Most of these circRNAs were generated by RNA circularization using back-splicing, and this process is like the alternative splicing event of the pre-mRNA. The same splicing signals were utilized by back-splicing and alternative splicing of the pre-mRNA. During the back-splicing, the junction site of circRNA is formed by a 3′,5′ phosphodiester bond using the downstream 5′ splice site and an upstream 3′ splice site, whereas, in the canonical alternative splicing, a 5′ splice site is joint with a downstream 3′ splice site (**Figure 3**) (58, 59). Many DNA viruses have split genes in their viral genomes, and most of the circRNAs derived from these viruses may form on reported mechanisms from eukaryotes, including inverted repeat sequences, exon skipping, RNA-binding proteins, repetitive complementary sequences, or m6A modification in pre-RNA (60–67). It is noted that most of the circRNAs are formed by these strategies after 3’ end processing (67). However, RNA viruses with small genomes and relatively fewer split genes in the viral genome. Therefore, the biogenesis of circRNAs from the RNA viruses may have distinct mechanisms that differ from DNA viruses.

Several RNA viruses have been confirmed with the RNA circularization in the virally infected cells. Most RNA viruses derived circRNAs were produced with the noncanonical mechanism. The circularization events of viral RNAs found in the BmCPV and GCRV showed that the splicing signals flanking the junction sites of vcricRNAs differed from the reported circRNAs formation in animals and some DNA viruses (36, 52, 53). However, viral circRNAs identified from the MERS-CoV, SARS-CoV-1, and SARS-CoV-2 showed that some were generated with back-splicing of the viral RNAs (54, 55). Viral circRNAs with less conservation than those generated from host pre-mRNA may have evolved rapidly (68). Therefore, we concluded that viral cricRNAs among different viral genomes might have different RNA circularization mechanisms, especially these viral circRNAs derived from the RNA viruses with segmented dsRNA genomes.

## The Potential Roles of Virally circRNAs

**Figure 4:**
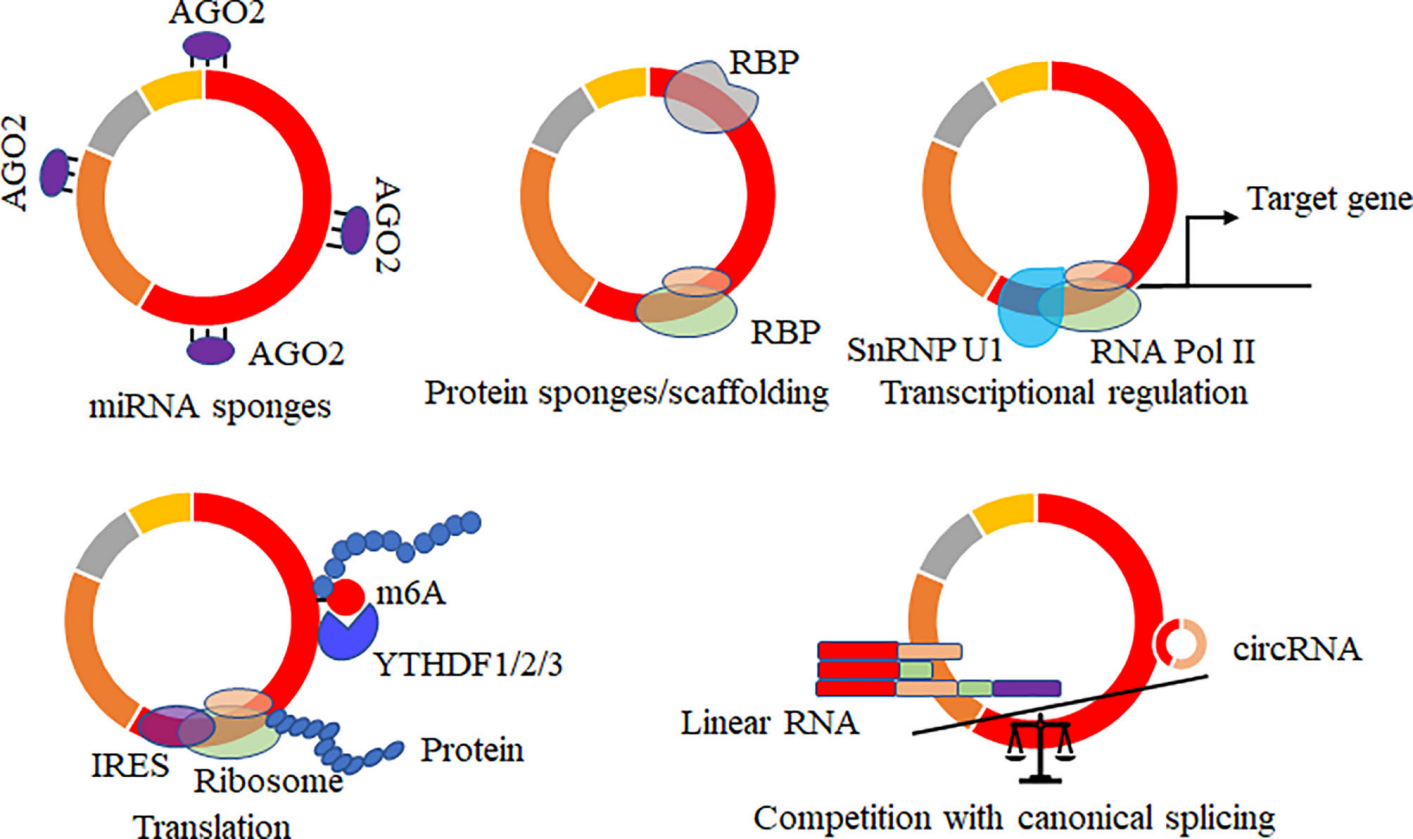
Biological functions of circRNAs. CircRNAs acted as microRNAs/RNA binding protein (RBP) sponges, mediated the alternative splicing and transcription process, regulated the expression levels of parental genes, translated proteins or small peptides, and mRNA trap that the formation of circRNA by back-splicing competes with canonical splicing of linear mRNA.

Increasing potential roles of circRNAs of hosts were reported, and several data showed that virally circRNAs with similar structures might have similar regulatory roles in various biological processes. The mainly reported roles of circRNAs acted as microRNAs (69–71)/RNA binding proteins (37, 72, 73) sponges, mediated the alternative splicing and transcription process (74–76), regulated the expression levels of parental genes (77), translated proteins or small peptides (36, 53, 78–80) (**Figure 4**). circRNA acts on the miRNA sponge, indirectly regulating the mRNA expression level of miRNA target genes and affecting various biological processes. circRNA generated by the parental sequence inevitably leads to a decrease in the level of linear RNA (25, 74). circRNA can also bind proteins or as the protein scaffolds to mediate the function of proteins. In addition, circRNAs containing partial internal ribosome entry sites (IRES) or m6A sites have been shown to translate proteins/small peptides in a cap-structure-independent manner (19, 36, 53).

Innate immunity acts as the first line of hosts to defend against pathogen invasion. During interactions and coevolution between the viruses and their hosts, the host’s immune system evolved various antiviral mechanisms, such as RNA interference, interferon, NF-κB-mediated, immune deficiency, stimulator interferon gene pathways, and Janus kinase/signal transducer and activator of transcription pathway. The generation of these antiviral signaling pathways restricts the process and speed of virus infection and the degree of disease in susceptible hosts. Meanwhile, various immune evasion strategies are evolved by viruses (81–84). Recently, a novel antiviral mechanism has been found that may evolve an immune regulatory mechanism to recognize exogenous circRNA (28). It has been established that RNA recognition receptor RIG-I is a receptor molecule that recognizes exogenous circRNA and activates the autoimmune effect pathway (85, 86). CircRNA forms a double-stranded RNA stem-loop structure that binds to the double-stranded RNA-dependent protein kinase (PKR). In normal cells, the antiviral PKR molecule is bound to the stem-loop structure of circRNA, avoiding the immune response caused by overactivation. During viral infection, RNase L cleaves the interacting circRNA and then degrades it. The released PKR participates in the antiviral immune process (24). When cells are infected with viruses, NF90/NF110, an antiviral dsRNA-binding protein originally located in the nucleus, will rapidly be transported to the cytoplasm, reducing mature circRNA that cannot be produced normally in cells. NF90/NF110 binds to viral mRNA and plays an antiviral role (87). There is a question about why viruses utilize their viral RNAs to produce viral circRNAs. We have predicted that these viral circRNAs may be generated by a host of unknown factors to suppress the viral overproliferation and avoid triggering innate immunity. A similar report said Simian virus 40 encodes miRNA to control its T-antigen to evade T cell immunity (88). Therefore, due to the similar structure and the conservation of the generation among species, we concluded that circRNAs derived from DNA or RNA viruses might have similar regulatory roles as these circRNAs encoded by the hosts.

## Possible Biogenesis Mechanism of Viral circRNAs and Host circRNAs

### Possible Biogenesis Mechanism of Viral circRNAs

CircRNA is a new type of RNA molecule derived from parental genes and mainly generated by a reverse splicing mechanism. This RNA molecule exists widely in nature and is highly conserved (47). Many reports on circRNA molecules encoded by natural organisms, from bacteria to mammals. A small subset of circRNA molecules with regulatory roles are associated with a verity of signaling pathways, among which the most in-depth studies are mainly focused on human cancer or tumor and other related studies. There are still some reports about circRNA produced by acellular organisms, predominantly in viruses. In the virally infected host cells, viral circRNA molecules may be widely produced in all types of viruses using various strategies. Some of these novel circRNAs could be assembled into virions (46). There are few reports on the formation mechanism of viral circRNAs. The circRNA production mechanism of DNA viruses with a large genome containing introns may be like that of circRNA production in higher animals by the back-splicing mechanism. However, some RNA viruses’ mechanism of circRNA production, which genomes have not contained introns, and the viral circRNAs derived from these viruses have not been well reported *in vitro* and *in vivo*. Several formation mechanisms for circRNAs in other species, including inverted repeat sequences, exon skipping, RNA-binding proteins, repetitive complementary sequences, or m6A modification in pre-mRNA, may be associated with the production of viral circRNAs from DNA viruses (60–67). However, the viral RNA circularization for circRNA was not comprehensively validated in some RNA viruses. In the SARS-CoV-2-infected Vero E6 cells, the homologous and reverse complementary sequences flanking the junction of sites of viral circRNAs may play vital roles in the frequency and the accuracy of viral RNA circularization (55).

### Possible Biogenesis Mechanism of Host circRNAs

The RNA circularization for circRNAs has been comprehensively reported, and too many RNA-binding proteins, cis-regulatory elements, trans-acting factors, and m6A modification were confirmed to take part in the host circRNA formation. Heterogeneous nuclear ribonucleoprotein M (HNRNPM) suppressed aberrant exon inclusion and circularization of transcripts in cells. HNRNPM preferentially interacted with GU-rich elements in the long flanking proximal introns in the mis-spliced linear and circular transcripts (89). Heterogeneous nuclear ribonucleoprotein L (HNRNPL) facilitated the generation of a novel oncogenic circARHGAP35 originating from the back-splicing of Exon2 and Exon3 of the *ARHGAP*35 gene (90). Long flanking and minimal repeats (<40 nt) in pre-mRNA-controlled circRNAs predominately after 3’ end processing. Heterogeneous nuclear ribonucleoprotein and serine-arginine proteins regulated the expression of circRNAs with a common back-splicing strategy (66). Heterogeneous nuclear ribonucleoprotein C (HnRNP C) controlled the expression of MERS-CoV-derived circRNAs (91).

Neuro-oncological ventral antigen 2 (NOVA2) related to splicing event globally promoted circRNA circularization using the NOVA2 binding sites in both flanking introns of circRNA loci (92, 93). Cannabinoid receptor type-1 (CB1) and fused in sarcoma (FUS) protein were involved in circCNOT6L circularization in testis (94). Splicing factor proline/glutamine-rich and non-POU domain-containing octamer-binding protein mediated the Alu-independent circRNA production (64). Cap-binding protein 80, C2HC zinc fingers superfamily protein, and flowering locus kh domain were related to the processes of splicing and the proper order of the exons, which expression levels were negative with the circRNA production (14). Serine and Arginine Rich Splicing Factor 10 (SRSF10) promoted circ-ATXN1 production by binding to the 5′-end and 3′-end of its pre-mRNA (95). Src-associated protein (Sam68) is a member of the signal transduction activator of the RNA family favored circRNA production *in vitro* and *in vivo via* binding in the proximity of intronic Alus in the pre-mRNA of Spinal Muscular Atrophy gene (96). Long and distinct repeat-rich intronic sequences and more reverse complementary motifs favored circular RNA biogenesis (97). Nudix Hydrolase 21 (NUDT21) as an RNA splice factor was confirmed to promote circRNA production, and the UGUA sequences in the pre-mRNAs were vital for circRNA circularization (98). Trans-acting factors such as RtcB ligase and the tRNA splicing endonuclease(TSEN) complex components played important roles in tricRNA formation (99).

Drosophila DExH/D-box helicase Hel25E and human homologs of Hel25E were related to the nuclear accumulation of long circRNAs. UAP56 (DDX39B) and URH49 (DDX39A) controlled the nuclear export of long and short circRNAs (100). RNA circularization was mediated by RNA binding protein Quaking (QKI) *via* binding upstream and downstream of exons (101). Several RNA binding proteins, FUS, Adenosine Deaminases Acting on RNA (ADAR), QKI, and Muscleblind (MBL), were associated with splicing regulation, RNA editing, and circRNAs production (32, 62, 94, 102). RNA-binding protein Trinucleotide repeat-containing 6A was responsible for circ0006916 formation *via* binding to the flanked intron region of the cognate linear transcript of circ0006916 (63). The RNA-binding protein FUS controlled circRNA production by back-splicing reactions (103).

The back-splicing occurred at m6A-enriched sites in male germ cells, and these circRNAs encompassed the m6A-modified start codons in their junction sites in large ORFs (67). Increasing data showed that intronic repetitive elements, complementary sequences from different introns, and RNA-binding proteins play crucial roles in forming circRNAs based on the canonical spliceosomal machinery (102, 104–106). In addition, the functional 3’ end processing signal was reported to be responsible for the RNA circularization, indicating that circRNAs were likely to form post-transcriptionally (65). The variety of circRNAs formation and the alternative circularization of RNA sequences will broaden the posttranscriptional regulation of RNAs in different species.

## Conclusions and Future Perspectives

**Figure 5:**
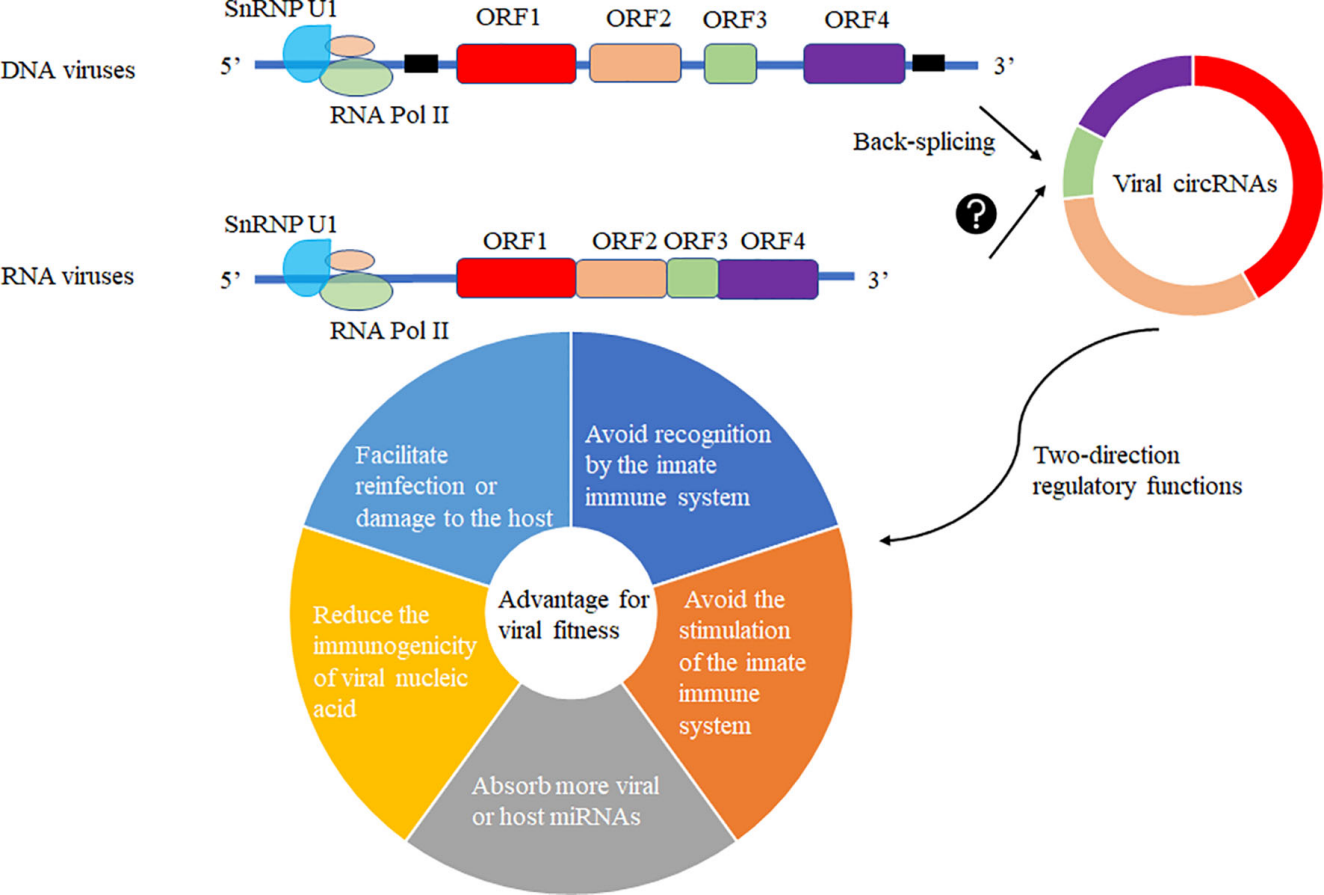
Viral circRNAs produced in the virus’s infected cells may have two-direction regulatory functions mediating the host or virus itself. The DNA viruses with coding regions (ORF) and noncoding regions and RNA viruses without noncoding regions among ORFs were selected to predict the production of viral circRNAs using different biogenesis. These viral circRNAs may have a variety of regulatory roles to host or the virus itself for fitness advantage to viruses.

The production of circRNA may be due to the fine regulation of the host cell to the genetic information of the virus so that its smaller genome carries more genetic information and regulates the immune system of the host cell or the life cycle of the virus to achieve mutual “reciprocity”. In the virally infected cells, viral circRNAs may be generated by the host cell machinery for its advantage or recruitment to suppress viral replication. Various viral circRNAs produced in the virus’s infected cells may have two-direction regulatory functions mediating the host or virus itself (**Figure 5**). Increasing functional viral circRNAs was confirmed, but there are still many mysteries about vcircRNA biogenesis.

Viruses invade host cells and release genetic material inside the cells. These genetic materials enter the nucleus for replication and proliferation. As the circRNA formation machinery is conservative in the cell, we can observe the existence of circRNA in lower animals to higher animals, which proves that the formation mechanism of circRNA is very conservative. The modification system may covalently modify the viral genetic material entering the nucleus in the host cell, such as m6A modification. Such modification will make the host immune system unable to distinguish whether it is its genetic material or foreign nucleic acid species, thus avoiding recognition by the innate immune system. The modified genetic species can be selectively spliced, recombined, and translated like the host genetic material. Another mechanism may be that foreign substances are circularized in host cells to reduce the levels of linear RNA and corresponding proteins, avoiding the stimulation of the innate immune system. Third, circularization may absorb more viral or host miRNAs that are not conducive to virus proliferation. Forth, the circularization of RNAs in host cells may reduce the immunogenicity of viral nucleic acid. Fifth, the circularization of RNAs may preserve the virus’s genetic material to facilitate reinfection or damage to the host (**Figure 5**).

As to these novel types of RNA molecules, more questions need to be answered:

(1) Whether multiple circRNAs are produced simultaneously or followed with cognate linear RNA transcripts?
(2) Can these circularizations of viral RNAs be inherited vertically in virally infected cells as regulatory elements to protect the host from reinfection?
(3) Whether viral circRNAs formation strategy will be manipulated by the host to avoid the degradation of viral RNAs?
(4) Whether these viral circRNAs are produced under certain circumstances, such as virus infection, different developmental stages, and other stimulates or other stimuli?
(5) Whether multiple viral circRNAs produced in the virally cells have a competitive interaction with other RNA transcripts, such as non-coding RNA, small RNAs, and genes between viruses and hosts?
(6) Whether the translation of viral circRNAs is the novel, unknown structural, and nonstructural proteins required for the viral life cycle?
(7) Whether these circRNAs can form a circRNA subgenome of viruses?
(8) Whether circRNAs are recruited by RNA binding proteins to form RNA virus replication regions?
(9) Why so small a virus genome can produce so many circRNAs?
(10) How do we balance the roles conducted by circRNAs and other non-coding RNAs or genes?
(11) Whether some viral circRNAs are pro-virions (immature virions)?
(12) Can some viral circRNAs fuse with the host or other viral circRNAs?

## Author Contributions

XZ and ZL wrote the draft. XH and CG reviewed and supervised the manuscript. XZ, ZL, CW, ZS and SS collected the data. All authors contributed to the article and approved the submitted version.

## Funding

This study was supported by the National Natural Science Foundation of China (31872424, 32072792, and 31972620) and the Priority Academic Program of Development of Jiangsu Higher Education Institutions. The funders had no role in study design, data collection, analysis, publishing decisions, or manuscript preparation.

## Conflict of Interest

The authors declare that the research was conducted in the absence of any commercial or financial relationships that could be construed as a potential conflict of interest.

## Publisher’s Note

All claims expressed in this article are solely those of the authors and do not necessarily represent those of their affiliated organizations, or those of the publisher, the editors and the reviewers. Any product that may be evaluated in this article, or claim that may be made by its manufacturer, is not guaranteed or endorsed by the publisher.
